# Resveratrol prevents inflammation-dependent hepatic melanoma metastasis by inhibiting the secretion and effects of interleukin-18

**DOI:** 10.1186/1479-5876-9-59

**Published:** 2011-05-12

**Authors:** Clarisa Salado, Elvira Olaso, Natalia Gallot, Maria Valcarcel, Eider Egilegor, Lorea Mendoza, Fernando Vidal-Vanaclocha

**Affiliations:** 1 Innoprot SL, Bizkaia Technology Park, Derio, Bizkaia, Spain; 2 University of the Basque Country, School of Medicine and Dentistry, Bizkaia, Spain; 3 Pharmakine Ltd, Bizkaia Technology Park, Bizkaia, Spain; 4 CEU-San Pablo University School of Medicine, Institute of Applied Molecular Medicine (IMMA), Boadilla del Monte, Madrid, Spain

## Abstract

**Background:**

Implantation and growth of metastatic cancer cells at distant organs is promoted by inflammation-dependent mechanisms. A hepatic melanoma metastasis model where a majority of metastases are generated via interleukin-18-dependent mechanisms was used to test whether anti-inflammatory properties of resveratrol can interfere with mechanisms of metastasis.

**Methods:**

Two experimental treatment schedules were used: 1) Mice received one daily oral dose of 1 mg/kg resveratrol after cancer cell injection and the metastasis number and volume were determined on day 12. 2) Mice received one daily oral dose of 1 mg/kg resveratrol along the 5 days prior to the injection of cancer cells and both interleukin-18 (IL-18) concentration in the hepatic blood and microvascular retention of luciferase-transfected B16M cells were determined on the 18^th ^hour. *In vitro*, primary cultured hepatic sinusoidal endothelial cells were treated with B16M-conditioned medium to mimic their *in vivo *activation by tumor-derived factors and the effect of resveratrol on IL-18 secretion, on vascular cell adhesion molecule-1 (VCAM-1) expression and on tumor cell adhesion were studied. The effect of resveratrol on melanoma cell activation by IL-18 was also studied.

**Results:**

Resveratrol remarkably inhibited hepatic retention and metastatic growth of melanoma cells by 50% and 75%, respectively. The mechanism involved IL-18 blockade at three levels: First, resveratrol prevented IL-18 augmentation in the blood of melanoma cell-infiltrated livers. Second, resveratrol inhibited IL-18-dependent expression of VCAM-1 by tumor-activated hepatic sinusoidal endothelium, preventing melanoma cell adhesion to the microvasculature. Third, resveratrol inhibited adhesion- and proliferation-stimulating effects of IL-18 on metastatic melanoma cells through hydrogen peroxide-dependent nuclear factor-kappaB translocation blockade on these cells.

**Conclusions:**

These results demonstrate multiple sites for therapeutic intervention using resveratrol within the prometastatic microenvironment generated by tumor-induced hepatic IL-18, and suggest a remarkable effect of resveratrol in the prevention of inflammation-dependent melanoma metastasis in the liver.

## Background

Individuals at high risk of metastasis from malignant tumors are a large group of patients that still does not receive an efficient treatment. The development of low-toxicity drugs that target molecular mechanisms promoting intravascular dissemination, microvascular arrest, and micrometastatic growth of cancer cells is becoming a feasible strategy to prevent adverse clinical effects of the metastatic disease in cancer patients. Because inflammation and oxidative stress have prometastatic implications at these subclinical stages of metastasis inception [1,2], agents that target specific genes and molecules that regulate these host responses to tumor-derived factors may become good anti-metastatic candidates for clinical translation.

Resveratrol (RVL) --a phytopolyphenol that occurs in grapes and various other fruits and medicinal plants [3]-- is a broad-spectrum anti-oxidant that inhibits the experimental development of several cancer types at diverse stages, metastasis included [4-5, for review see 6], and at relatively non-toxic doses. Not surprisingly, the effects exerted by RVL are consistent with its capacity to interact with molecular targets that are relevant during carcinogenesis, but also during metastasis. Specifically, RVL inhibits STAT3 and NF-kappaB-dependent transcription [6,7], Bcl-xL expression [8] and hypoxia-induced HIF-1alpha and VEGF [9], while it activates p53 [10] and TRAIL expression [11]. Moreover, nitric oxide initiates the progression of human melanoma via a feedback loop involving the apurinic/apyrimidinic endonuclease-1/redox factor-1, which is also inhibited by RVL [12]. However, much work needs to be done for a more complete understanding of its mechanisms of action and therefore, for a better assessment of its anti-tumor efficacy.

The experimental hepatic colonization of B16 melanoma (B16M) is a unique model for determining therapeutic intervention sites of natural antioxidant products, such as RVL, in the prometastatic microenvironment created in the liver by tumor-induced microvascular inflammation. Intrasplenic and left-cardiac ventricle B16M cell injection routes are followed by formation of hepatic metastases, the majority of which are proinflammatory-cytokine dependent, as shown in IL-1beta- and IL-1 converting enzyme-deficient mice [13], and with recombinant IL-1 receptor antagonist [14] and IL-18 binding protein treatments [15]. Consistent with melanoma metastasis regulation by proinflammatory cytokines, response of primary cultured hepatic sinusoidal endothelium (HSE) to B16M cell soluble factors remarkably increased cancer cell adherence to tumor-activated endothelium. This is due to a sequential process involving TNF-alpha-dependent IL-1beta, which in turn induced IL-18 to upregulate VCAM-1 via hydrogen peroxide (H_2_O_2_) [16]. Moreover, blockade of VCAM-1 with specific antibodies prior to B16M cell injection significantly decreased hepatic retention of B16M cells and metastasis development [17]. Because VCAM-1 expression is oxidative stress-inducible, *in vivo *administration of recombinant catalase resulted in a complete abrogation of both enhanced VCAM-1 expression by HSE cells obtained from tumor-injected mice and increased B16M cell adhesion to those HSE [16]. The pivotal position of H_2_O_2 _in this metastasis model was further supported by the fact that incubation of HSE cells with non-toxic concentrations of H_2_O_2 _also directly enhanced *in vitro *VCAM-1-dependent B16M cell adhesion without inflammatory cytokine mediation [16]. B16M cells also responded to hepatic-derived IL-18 by enhanced proliferation [15] and increased adhesion to HSE via a VLA-4-dependent mechanism [17].

In this study, we investigated the effect of RVL on the microvascular phase of the hepatic metastasis process of B16M cells. First, the effects of RVL on the capillary arrest and early metastatic growth of intrasplenically-injected B16M cells were studied *in vivo*. Second, because IL-18 regulates melanoma metastasis occurrence via VLA-4-dependent B16M cell adhesion to HSE, the effects of RVL on IL-18 secretion and VCAM-1 expression by hepatic sinusoidal cells, and on the cancer cell response to IL-18 were studied *in vitro*.

Our results showed that RVL remarkably inhibited both hepatic microvascular retention and metastatic development of B16M cells. *In vitro*, RVL completely abrogated the melanoma cell adhesion to tumor-activated HSE operated via VLA-4/VCAM-1 interaction. Our results also showed that the antimetastatic effect of RVL was exerted in this model through an efficient blockade of IL-18 effects, which was secreted by tumor-activated hepatic tissue and promoted VLA-4-dependent melanoma cell adhesion and proliferation via hydrogen peroxide-dependent NFkappaB activation. These findings suggest that RVL can act as a powerful inhibitor in the prometastatic microenvironment of hepatic inflammation generated by tumor-induced host IL-18.

## Materials and methods

### Cells and culture conditions

B16M cells (B16F10 subline) were cultured in DMEM supplemented with 10% FCS and penicillin-streptomycin (Sigma Chemicals Co., St. Louis, MO) [10]. B16M-conditioned medium (B16M-CM) was obtained from subconfluent cells cultured for 12 hours as previously described [13].

### Hepatic metastasis model and treatment schedule

Syngeneic C57BL/6J mice (male, 6-8 weeks old) were obtained from IFFA Credo (L'Arbreole, France). Animal housing, care, and experimental conditions were conducted in conformity with institutional guidelines, in compliance with the relevant national and international laws and policies. Hepatic metastases were produced by intrasplenic injection of B16 cells as previously described [14]. Mice were killed on the twelfth day afterwards. Liver tissue was processed for histology [14]. Fifteen 4 μm-thick tissue sections of formaldehyde-fixed liver (five groups, separated 500 μm) were stained with H&E. An integrated image analysis system (Olympus Microimage 4.0 capture kit) connected to an Olympus BX51TF microscope was used to quantify the number, average diameter, and position coordinates of metastases. Percentage of liver volume occupied by metastases and metastasis density (foci number/100 mm^3^) were also determined [14]. In order to study the effect of RVL (Sigma-Aldrich Chemicals Co, St Louis, MO) on hepatic metastasis development, some mice (10 per group) received 1 mg/kg/day RVL dissolved in ethanol (5%), via intragastric tube, from day 1 to 12. Control mice received the same volume of vehicle. Each experiment was carried out three times.

### Quantitative assay on hepatic retention of circulating melanoma cells

B16M cells were stably transfected with a construct containing the *Photinus pyralis *luciferase gene coding sequence under transcriptional control of the cytomegalovirus promoter and the neomycin resistance gene [16]. Three hundred thousand viable luciferase-transfected B16M cells were intrasplenically injected into C57BL/6J mice (*n *= 30). Some mice received 1 mg/kg/day RVL five days prior to B16M-Luc cell injection. All mice were killed 18 hours later, and livers were analyzed for luciferase activity (Promega Co., Madison, WI) as described previously [16].

### Isolation of hepatic sinusoidal cells and enriched primary culture of endothelial cells

HSE cells were isolated from syngeneic C57BL/6J mice (male, 6-8 weeks old) identified, and cultured as described previously [16]. Briefly, isolated mouse liver cells were obtained by two-step collagenase perfusion. A non-parenchymal liver cell fraction was further purified by centrifugation in a Percoll^® ^gradient (Amersham; Uppsala, Sweden). Kupffer cells were then removed by selective adherence to plastic substrate, HSE cells were phenotypically characterized by flow cytometry analysis with specific antibodies against CD31 (PECAM-1 Sigma, St Louis), HLA class II and CD40, (both from BD Biosciences); CD106 (VCAM-1) and CD14 (both fromBD Pharmingen, San Diego, CA), smooth muscle alpha actin (ASMA, Sigma-Aldrich, St Louis, MO). HSE cells were seeded at 2 × 10^5 ^cells/well in RPMI-1640 culture medium (Sigma Chemicals, St. Louis, MO) supplemented with 10% FCS (Life Technologies, Gaithersburg, MD) onto 24-well tissue culture plates pre-coated with type I collagen solution (0.03 mg/ml) (Collagen Biomaterials, Palo Alto, CA) and allowed to spread for 45 min at 37°C and 5% CO_2_.

### Enzyme immunoassay of IL-18 concentration in hepatic blood and supernatants of cultured cells

Serum samples were obtained from hepatic (suprahepatic vein) blood of adult male C57/B1/6J mice 18 hours after intrasplenic injection of B16M cells. Some mice received (1 mg/kg/day) RVL from day 1 to 5 via intragastric tube prior to melanoma cells injection. Primary cultured HSE cells were incubated in the presence of absence of 2.5 μM RVL or recombinant VEGF antibody for 30 min, after which B16M-CM or 10 ng/ml of recombinant VEGF were added. Eight hours later, the supernatant from the treated HSE cells were collected. IL-18 concentration in serum from hepatic blood or in culture cell media was detected using a competitive enzyme immunoassay (R&D Systems, Minneapolis, MN).

### Immunohistochemical analysis of p65 nuclear translocation

B16M cells (1 × 10^4 ^cells/well) were grown on 8 μm-chamber glass slides. Cells were serum-starved for 24 h and treated with IL-18 (100 ng/ml) for 30, 60 and 120 min. In some experiments, cells received 2.5 μM RVL 30 min prior to IL-18 treatment. Once treatment time had finished, cells were fixed in 4% formaldehyde (in PBS) for 30 min at room temperature and permeabilized in 1% SDS for 10 min. Non-specific binding was blocked for 1 hour with 10% bovine serum in PBS buffer. Cells were incubated with 1.5 μg/ml rabbit anti-p65 polyclonal antibody (Santa Cruz Biotechnology Inc., Santa Cruz, CA) for 1 h at room temperature. Further wash steps removed unbound antibody prior to the addition of the secondary Alexa 594 goat anti-rabbit antibody. Images were acquired on a BD Pathway™ Bioimager.

### Western blot analysis of p65 in nuclear fractions

Subconfluent cultures of B16M cells were treated as above described for 60 min. Then, they were harvested and incubated in lysis buffer (10 mM HEPES (pH 7.9), 10 mM KCl, 0.1 mM EDTA, 0.1 mM DTT, 0.1% Nonidet P-40 and 0.5 mM PMSF) for 20 minutes on ice. The crude nuclei were collected by centrifugation, further incubated in 20 mM HEPES (pH 7,9), 0.4 mM NaCl, 1 mM EDTA, 1 mM DTT, 1 mM phenylmethylsulfonyl fluoride (PMSF) for 20 minutes on ice and clarified by micro-centrifugation and frozen. Fifty micrograms of nuclear extracts were resolved on 12% of SDS-PAGE and p65 protein was analyzed by Western blot using rabbit polyclonal antibody (Santa Cruz Biotechnology, Santa Cruz, CA). Lamin-B expression was used as loading control.

### Western Blot analysis of VCAM-1

Freshly isolated HSE cells were seeded onto 24-well plates for 24 hours. Then, they were cultured for 12 hours in the presence of basal medium, B16M-CM or 1 ng/ml IL-18. In some experiments, HSE cells received 2.5 μM RVL 30 min prior to B16M-CM or IL-18 treatment. HSE cells were disrupted in 50 mM Tris (pH 7.5), 150 mM NaCl, 1% NP40, 0.5% deoxycholic acid, 0.1% SDS, 2 mM EDTA, 10 mM NaF, 10 μg/ml leupeptin, 20 μg/ml aprotinin, and 1 mM PMSF. Then, 40 μg of protein from cell lysates were separated by 12% SDS-PAGE followed by Western analysis using rat anti-mouse VCAM-1 monoclonal antibody and β-tubulin (both from Santa Cruz Biotechnology, Santa Cruz, CA) and the appropriate secondary antibodies.

Blot was imaged using a Syngene G-Box gel imaging system (Synoptics Ltd., Cambridge) and band density analyzed using Gene Tools analysis software.

### Measurement of H_2_O_2 _production from B16M cells *in vitro*

B16M cells were cultured in DMEM without phenol red with 20 μmol/L 2', 7'-dichlorofluorescein-diacetate (DCFH-DA) as described [12]. H_2_O_2 _produced by incubated cells oxidizes DCFH to the highly fluorescent DCF so that fluorescence intensity is directly proportional to the amount of H_2_O_2 _produced by the cells. DCF fluorescence was recorded at 485/22-nm excitation and 530/25-nm emission filters. Non-DCFH-DA-incubated cells were used to determine basal autofluorescence. B16M cells were treated with 1 ng/ml IL-18, for 2 hours. In some experiments B16M cells were incubated with 2.5 μM of RVL 30 minutes prior to IL-18 addition. H_2_O_2 _production per well was quantified in arbitrary fluorescence units after subtracting basal autofluorescence.

### Proliferation assay

B16M cells were cultured overnight in DMEM plus 10% FCS. Then, they were incubated for 72 hours in the presence of 0.1 ng/ml IL-18 supplemented with 0.5% FCS. Control cells received the same culture medium without the cytokine. In some experiments, 2.5 μM RVL was added to control and IL-18-treated B16M cells. After 48 hours incubation, B16M cell proliferation was measured using sulforhodamine 101 protein assay, as described previously [15].

### B16M cell adhesion assay to primary cultured hepatic endothelial cells

B16M cells were labeled with 2', 7'-bis-(2-carboxyethyl)-5,6-carboxyfluoresceinacetoxymethylester solution (Molecular Probes, Eugene, OR) and added to primary culture of HSE cells (2 × 10^5 ^cells/well). Eight minutes later, wells were washed three times with fresh medium. The number of adhering cells was determined using a quantitative method based on a previously described fluorescence measurement system [14]. HSE were treated with B16M-CM for 6 hours prior to the addition of B16M cells. In some experiments, HSE cells received 2.5 μM RVL or vehicle 30 minutes prior to B16M-CM. In other experiments, B16M cells were pretreated with IL-18 for 6 hours prior to the adhesion assay. In this case, B16M cells received 2.5 μM RVL or vehicle 30 minutes before IL-18.

### Statistical analyses

Data were expressed as means ± SD. Statistical analysis was performed by SPPS statistical software for Microsoft Windows, release 6.0 (Professional Statistic, Chicago, IL). Homogeneity of the variance was tested using the Levene test. If the variances were homogeneous, data were analyzed by using one-way ANOVA test with Bonferroni's correction for multiple comparisons when more than two groups were analyzed. For data sets with non-homogeneous variances, ANOVA test with Tamhane's posthoc analysis was applied. Individual comparisons were made with Student's two-tailed, unpaired *t *test (program Statview 512; Abacus Concepts, Inc., for Macintosh). The criterion for significance was P < 0.01 for all comparisons.

## Results

### Resveratrol inhibits hepatic seeding and growth of metastatic melanoma cells

**Figure 1:**
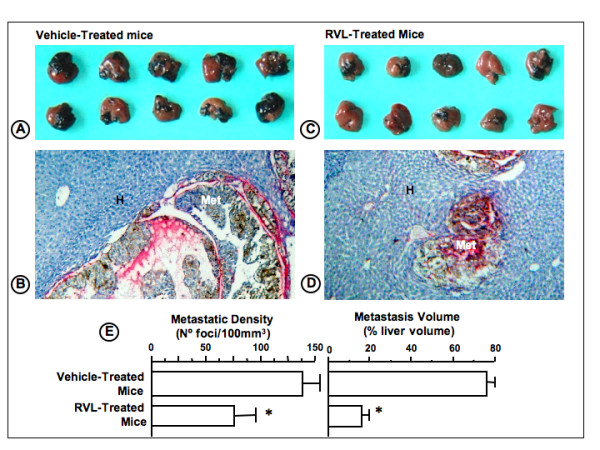
**Effect of resveratrol on hepatic metastasis development of intrasplenically-injected B16M**. Mice were intrasplenically injected with B16M cells and 1 mg/kg/day RVL was administered via intragastric tube, from day 1 to 12. A and C) Representative picture of livers from vehicle- and RVL-treated mice obtained on the day 12 after cancer cell injection. Metastases are visible as black melanotic nodules. B and D) Representative microscopic pictures of hepatic tissue sections from vehicle- and RVL-treated mouse livers. Tumor-affected hepatic tissue in blue (H) and hepatic metastases in brown due to melanogenic cells (Met). Anti-smooth muscle-alpha antibodies were used to immunohistochemically stain metastasis-associated stromal cells (in red). E) Metastatic volume and number in untreated and RVL-treated mice (n = 10 per group), as percentage of liver occupied by metastases and number of foci per 100 mm^3^, respectively. Histograms represent average values ± SD of 3 independent experiments. *Differences were statistically significant with respect to vehicle-treated mice (P < 0.01) according to Bonferroni and post-hoc. ANOVA test.

**Figure 2:**
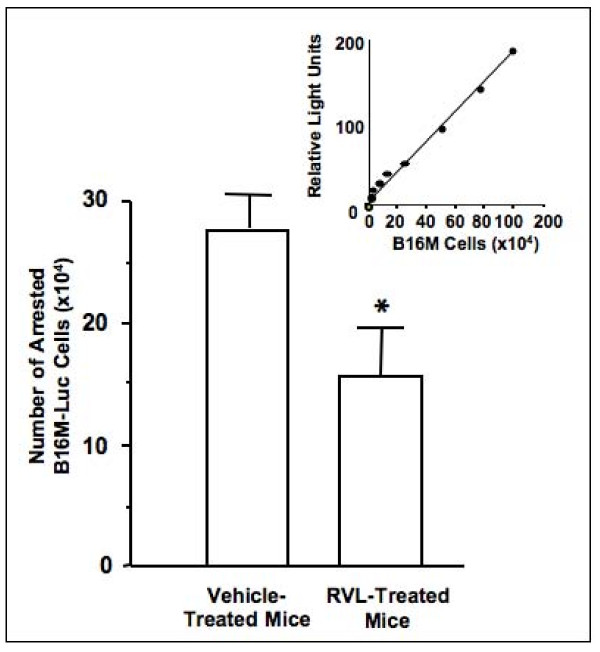
**Effect of resveratrol on melanoma cell retention in the hepatic microvasculature of intrasplenically-injected B16M**. RVL was administrated via intragastric tube (1 mg/kg/day) during 5 days prior to 3 × 10^5 ^B16M-Luc cell intrasplenic injection. After 18 hours livers were removed and light production was measured as described in Methods. Light emission values were expressed as relative light units and the number of arrested B16M-Luc cells was calculated on the basis of a standard curve relating specific relative light units to B16M-Luc cell number. Data represent average values ± SD of two separate experiments. *Differences were statistically significant (P < 0.01) to respect to vehicle-treated mice, by Student's two tailed, unpaired t test.

RVL given orally at 1 mg/kg from day 1 to 12 after B16M cell injection reduced hepatic metastasis volume by 75% as compared to control mice treated with vehicle (Figure 1A-1E). Antimetastatic activities of RVL did not involve any secondary effect that might compromise animal survival. The same treatment schedule also significantly (P < 0.01) decreased metastasis number by 50% (Figure 1E). Moreover, majority of metastases developed in RVL-treated mice were on average significantly smaller (by 60%) than those developed in vehicle-treated mice. Therefore, B16M cells predominantly colonized hepatic tissue through RVL-sensitive mechanisms. Consistent with RVL-dependent decrease in hepatic metastasis number, oral administration of the same daily dose of RVL to animals during 5 days prior to cancer cell injection, led to a statistically significant (P < 0.01) hepatic retention decrease by 45% of luciferase-transfected B16M cells (Figure 2.). Moreover, although anti-metastatic effects of RVL affected 50% of metastases, hepatic metastasis volume decreased by 75%, indicating that either RVL-resistant metastasis had a slower growth rate than RVL-sensitive ones, or that metastasis implantation by RVL-resistant mechanism still had a RVL-sensitive growth mechanism.

Consistent with previous reports [18], our histologic study (Figure 1B and 1D) showed that hepatic melanoma metastases from vehicle-treated mice were predominantly of sinusoidal-type, containing a rich network of intratumoral microvessels supported by sinusoidal-derived myofibroblasts; while a minority were of portal-type, containing a lower density of intratumoral microvessels, mainly supported by portal tract-derived myofibroblasts. Interestingly, RVL decreased by 80% sinusoidal-type metastasis number, while it did not alter the number of portal-type metastasis, indicating that RVL-sensitive metastases were of sinusoidal-type (E Olaso, C Salado, B Arteta, A Lopategi, F Vidal-Vanaclocha: Resveratrol inhibits the proangiogenic response of hepatic sinusoidal cells to tumor-derived soluble factors during liver metastasis by colon carcinoma, submitted).

### Resveratrol inhibits hepatic secretion of IL-18 induced by infiltrating metastatic melanoma cells

**Figure 3:**
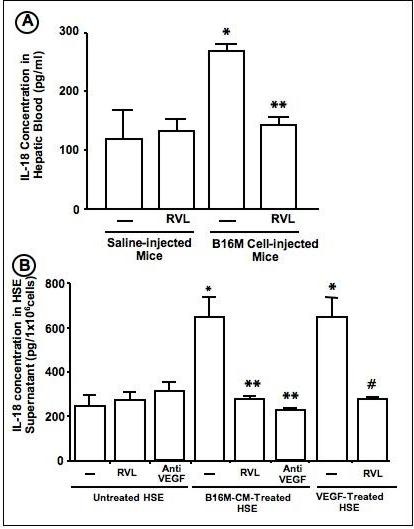
**Effect of RVL on IL-18 secretion from tumor-activated HSE cells *in vivo *and *in vitro***. A) IL-18 levels were determined in serum samples obtained from suprahepatic vein blood on the 18^th ^hour after intrasplenic injection of 3 × 10^5 ^B16M cells. Some mice received 1 mg/kg/day RVL via intragastic tube 5 days prior to cancer cells injection. B) Primary cultured HSE cells were incubated in the presence or absence of 2.5 μM RVL or recombinant VEGF antibody for 30 min, and then with either B16M-CM or 10 ng/ml recombinant murine VEGF. Eight hours later, the supernatants from treated HSE cells were collected and IL-18 concentration determined by ELISA. Data represent the average values ± SD of 3 independent experiments. *Differences were statistically significant (P < 0.01) to respect to vehicle-treated mice, by Student's two tailed, unpaired t test.

Previously, we reported that IL-18 increased in hepatic venous blood over basal level during the sinusoidal inflammation associated with liver-infiltrating cancer cells [13,15]. A similar 5 day-RVL pretreatment schedule as above also completely abrogated the augmentation of IL-18 concentration in the hepatic blood obtained 18 hours after B16M cell injection into treated mice, without affecting the physiological levels of this cytokine in mice not injected with tumor cells (Figure 3A). In order to discard that the increase in plasma IL-18 might reflect a decreased hepatic metabolism of this cytokine subsequent to the metastasis process [19], we next determined the effect of RVL on IL-18 secretion from tumor-activated hepatic sinusoidal cells. Primary cultured HSE cells received either RVL or vehicle 30 min prior to being treated with B16M-CM for 8 hours and the level of IL-18 was measured in the supernatant by ELISA. As shown in Figure 3B, RVL abolished the augmentation of IL-18 concentration in B16-CM-treated HSE cells, while it did not affect basal IL-18 concentrations in the supernatant of untreated primary cultured HSE cells.

Previously, we showed that the pro-adhesive effect of IL-18 on tumor-activated HSE cells was VEGF-dependent [20]. Herein, we showed that anti-murine VEGF antibody completely abrogated IL-18 secretion from tumor-activated HSE cells and that RVL abolished IL-18 production from VEGF-activated HSE cells. Therefore, RVL inhibited IL-18 secretion from tumor-activated HSE cells through the specific inhibition of tumor-derived VEGF on HSE (Figure 3B).

### Resveratrol inhibits IL-18-induced VCAM-1 expression on tumor-activated hepatic sinusoidal endothelium, preventing microvascular adhesion of melanoma cells

**Figure 4:**
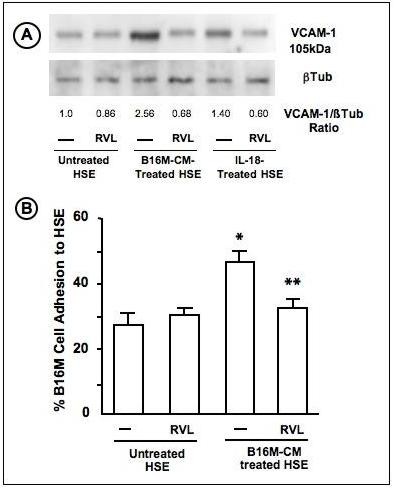
**Effect of resveratrol on hepatic sinusoidal endothelial cell response to tumor-derived factors**. A) Representative experiment on the effect of RVL and IL-18 on VCAM-1 protein expression by tumor-activated HSE. Freshly isolated and primary cultured murine HSE cells were incubated for 30 min with 2.5 μM RVL or vehicle, and next with B16M-CM or 1 ng/ml IL-18 overnight. Then, cell lysates were obtained and VCAM-1 protein was analyzed by Western Blot. Quantification of VCAM-1 expression was normalized to that of β-tubulin. B) Effect of RVL on B16M cell adhesion to untreated and B16-CM treated HSE *in vitro*. Cultured HSE were incubated with basal medium and B16M-CM for 6 hours. In some wells, 2.5 μM RVL was added 30 minutes before B16M-CM. Then, cell adhesion assay was performed as described in Methods. The results are average values ± SD of 3 separated experiments, each in triplicate (n = 9). Differences in the percentage of adhering cells with respect to untreated HSE (*) or B16M-CM treated ESH (**) were statistically significant (P < 0.01) by ANOVA and Bonferoni's post-hoc test.

Because IL-18 promotes the adhesion of B16 melanoma cells to the hepatic microvascular endothelium via VCAM-1-dependent mechanism [13,15], we next studied the effect of RVL on the VCAM-1 expression level in primary cultured hepatic endothelial cells given either recombinant murine IL-18 or B16M-CM. As shown in Figure 4A, pretreatment of HSE cells with RVL abrogated VCAM-1 expression increase in HSE cells given either IL-18 or B16M-CM. Consistently, RVL also abolished the adhesion of B16M cells to HSE cells given the same B16M-CM for 6 hours prior to adhesion assays (Figure 4B).

### Resveratrol inhibits adhesion- and proliferation-stimulating effects of IL-18 on metastatic melanoma cells

**Figure 5:**
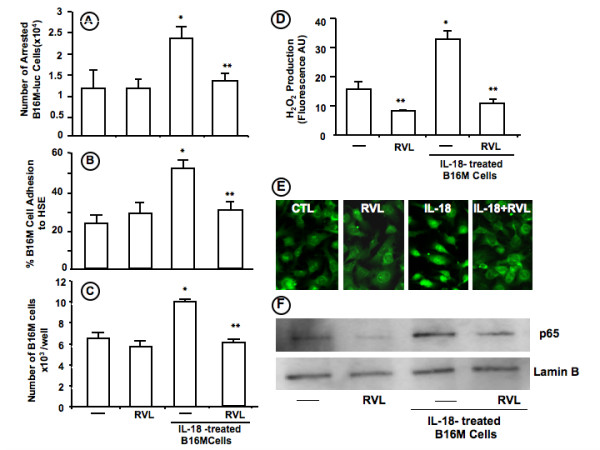
**Effect of RVL on B16M cell response to IL-18**. B16M cells, either untreated or pre-treated for 30 minutes with 2.5 μM RVL, were incubated with 1 ng/ml IL-18 for different time periods. A) Effect of RVL on B16M-Luc cell retention in hepatic microvasculature. Mice were intrasplenically-injected with either treated or untreated B16M-Luc cells and livers were analyzed after 18 hours. Light emission values were determined as described in Methods. The number of arrested B16M-Luc cells was calculated on the basis of a standard curve relating specific relative light units to B16M-Luc cell number. B) Effect of RVL on B16M cell adhesion to HSE. The percentage of adherent B16M cells was determined as described in Material and Methods. C) Effect of RVL on B16M cell proliferation. B16M cell proliferation was analyzed by sulforhodamine-101-based fluorometric assay. D) Effect of RVL on H_2_O_2 _production by B16M-Cells. H_2_O_2 _production was expressed as DCF fluorescence values (in fluorescence arbitrary units). E and F) Inhibitory effect of Resveratrol on IL-18-induced NF-κB activation. B16M cells were serum-starved for 24 h and treated with IL-18 (100 ng/ml) from 30- to-120 min. In some experiments, cells received 2.5 μM RVL 30 min before assays. Results shown are representative of the experiment after 60 min of treatment. NF-κB nuclear translocation was detected by immunofluorescent staining with anti p-65 antibody. (E). Western analysis was also performed with anti-p65 antibody and Lamin B as a loading control for the nuclear fraction (F). Every assay was done in triplicate and repeated three times. Data represent average values ± SD. *Differences were statistically significant with respect to cells in basal medium (P < 0.01) according ANOVA test.

As assessed by RT-PCR and flow cytometry, B16M cells expressed IL-18 receptor alpha under basal culture conditions [15,17]. Therefore, together with proinflammatory effects of IL-18 on HSE cells, melanoma cell function might also be affected by HSE-derived IL-18 in the microenvironment of tumor-activated liver [13]. As shown in Figure 5A, *in vitro *treatment with RVL completely abrogated the hepatic microvascular arrest augmentation induced in B16M cells given 1 ng/ml IL-18 for 6 hours prior to their intrasplenic injection into normal mice. The same treatment schedule with RVL also prevented both B16M cell adhesion to primary cultured HSE cells and B16M cell proliferation induced in IL-18-pretreated B16M cells, without affecting these functional activities in basal condition-cultured B16M cells (Figure 5B and 5C). Because IL-18 regulates H_2_O_2 _production from B16M-CM-treated HSE cells [16], we next studied the effect of RVL on H_2_O_2 _production from IL-18-treated B16M cells. Interestingly, B16M cells given 1 ng/ml IL-18 for 2 hours significantly (P < 0,01) increased their H_2_O_2 _production. However, this cytokine-induced oxidative reaction was completely neutralized when B16M cells received RVL 30 min prior to IL-18 (Figure 5D). Moreover, NF-kB activation is a known pathway for attenuating cancer cell response to inflammatory mediators. Therefore, we examined the nuclear translocation of p65, the transcriptionally active subunit of NF-kB, in cultured B16M cells given recombinant murine IL-18 (Figure 5E and 5F). First, as shown immunofluorescence using an anti-p65 antibody (Figure 5E), p65 remained cytosolic under basal conditions and no change was observed when basal condition-cultured cells received RVL. In contrast, a 60 min stimulation with IL-18 (100 ng/mL) remarkably induced p65 translocation into the nucleus. P65 translocation persisted for 120 min (data not shown). However, addition of RVL to IL-18-treated cells completely abrogated p65 translocation (Figure 5E). Second, similar results were obtained when nuclear extracts from same experimental conditions as above were analyzed for p65 protein expression by Western blot (Figure 5F). Our data therefore indicates that IL-18 activates NF-kB in B16M cells and that RVL can efficiently inhibits this activation.

## Discussion

We demonstrated that resveratrol prevented inflammation-dependent hepatic melanoma metastasis by inhibiting both secretion of IL-18 from tumor-affected liver and effects of hepatic IL-18 on melanoma cells. Daily treatment with RVL significantly inhibited murine melanoma metastasis occurrence and development. Consistent with these effects, oral administration of RVL during 5 days prior to melanoma cell injection inhibited both IL-18 augmentation in the hepatic blood and melanoma cell retention in the hepatic microvasculature of melanoma cell-injected mice. This was further supported by *in vitro *experiments where RVL also inhibited IL-18 secretion, VCAM-1 expression and melanoma cell adhesion to tumor-activated HSE cells, three interrelated events regulating the microvascular arrest of circulating melanoma cells in the liver [2]. Our results also revealed that RVL blocked both *in vitro *VLA-4-dependent microvascular adhesion and proliferation in IL-18-treated melanoma cells, suggesting that RVL may also exert its antimetastatic effect by preventing melanoma cell response to endogenous hepatic IL-18. Not surprisingly, both adhesion and proliferation were upregulated in IL-18-treated melanoma cells via hydrogen peroxide-dependent NF-kappaB activation, which was inhibited by RVL in this tumor model. Therefore, our findings provide evidence that RVL inhibited IL-18 secretion from tumor-activated hepatic tissue, and subsequently, IL-18 effects on both host and cancer cells, which prevented the inflammation-dependent metastasis class in the prometastatic microenvironment created by tumor-induced IL-18 in the liver.

Previous studies have already reported about anti-metastatic effects of RVL on various rodent and human solid tumors [5,6,21-23]. However, specific therapeutic intervention sites of RVL along metastasis process are still unclear. Herein, a hepatic melanoma metastasis model--where majority of metastases are generated via IL-18-dependent inflammatory mechanisms [13,15]--, served to demonstrate that RVL specifically interferes with inflammation-dependent metastases that develop in the liver through oxidative stress-mediated mechanisms. It has already been reported that RVL has beneficial effects in pathogenic conditions of the liver that involve an overproduction of inflammatory cytokines, such as cholestasis [24], alcohol injury [25], and LPS [26]. More importantly, it has also been shown that RVL suppresses oxidative stress and inflammatory response in diethylnitrosamine-initiated rat hepatocarcinogenesis [5,6]. However, at the moment RVL has not yet been reported as a potential inhibitor of prometastatic effects of hepatic inflammation created in the liver microenvironment by tumor-induced IL-18.

IL-18 is a proinflammatory cytokine that increases in the blood of the majority of cancer patients and that has been associated with disease progression and, in some cancer types, even with metastatic recurrence, poor clinical outcome and survival [17]. While recombinant IL-18 has very limited therapeutic activity as a single agent in patients with metastatic melanoma [27], preclinical studies have shown that IL-18 binding protein inhibits hepatic and lung metastases in murine models [15,28]. This is further supported by studies revealing that IL-18-dependent mechanisms promote immune escape [29], microvascular adherence [13], resistance to UVB-induced apoptosis [30], and angiogenesis [18,31]. Therefore, the fact that RVL completely abrogated the increase of hepatic blood IL-18 induced by tumor-derived factors suggests the potential use of RVL as a hepatic metastasis chemopreventive agent in cancer patients at high risk of hepatic metastasis, such as those suffering from a malignant uveal melanoma.

IL-18 is also a major IFN-gamma-inducing factor and both IL-18 and IFN-gamma act together in the host response to infection, but also in the pathogenesis of acute hepatic injury [32]. In our model, abrogation of tumor-induced hepatic IL-18 did not involve any decrease of IFN-gamma level, which also significantly increased in the hepatic blood of same animals (data not shown). This suggests that IL-18-independent pathways were also operating in the induction of IFN-gamma secretion during tumor metastasis development in this model.

Previously, we have reported that liver-infiltrating B16M cells activated their adhesion to HSE cells through a sequential process involving TNF-alpha-dependent IL-1beta, which in turn induced IL-18 to up-regulate VCAM-1 via H_2_O_2 _[13,16]. The pivotal position of IL-18-induced H_2_O_2 _was further supported by the fact that incubation of HSE cells with nontoxic concentrations of H_2_O_2 _directly enhanced VCAM-1-dependent B16M cell adhesion *in vitro *without pro-inflammatory cytokine mediation, which emphasizes the key role of oxidative stress in the pathogenesis of IL-18-dependent hepatic metastasis [16]. Our current results show that RVL abolished H_2_O_2 _production from IL-18-treated melanoma cells. This has implications in several mechanisms: first, because it prevents oxidative stress-dependent VLA-4 integrin activation in melanoma cells; second, because it prevents nuclear translocation of NFkappaB, which is oxidative stress-dependent as well; and third, because it blocks IL-18 receptor-expressing melanoma cell subpopulation enlargement [17].

RVL also decreased metastasis number by 50%, suggesting that hepatic colonization of this murine melanoma occurred via RVL-sensitive and RVL-resistant mechanisms. However, hepatic metastasis volume decreased by 75%, indicating that either RVL-resistant metastases had a slower growth rate than RVL-sensitive ones, or that most of metastasis implantation by RVL-resistant mechanism still had a RVL-sensitive growth mechanism. According to our results, RVL-dependent metastatic growth inhibition it may depend in part on direct anti-proliferative effects of RVL on IL-18-dependent melanoma cells. It also may be due to a decrease of angiogenic parameters in hepatic B16M metastases from RVL-treated mice (E Olaso, C Salado, B Arteta, A Lopategi, F Vidal-Vanaclocha: Resveratrol inhibits the proangiogenic response of hepatic sinusoidal cells to tumor-derived soluble factors during liver metastasis by colon carcinoma, submitted). In this case, the mechanism appears to depend on the remarkable inhibitory effect of RVL on the proangiogenic effects of tumor-activated hepatic myofibroblasts [18].

**Figure 6:**
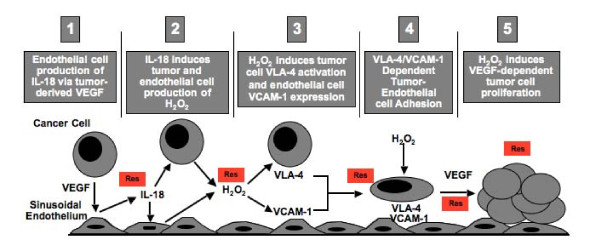
**Antimetastatic intervention sites for resveratrol (RVL) in the inflammatory microenvironment generated by tumor-activated hepatic sinusoidal endothelial cells**.

In summary, this study uncovers multiple therapeutic intervention sites for RVL in the inflammatory microenvironment of tumor-activated hepatic sinusoids occurring prior to metastasis development (Figure 6). First, RVL inhibited hepatic secretion of IL-18 induced by liver-infiltrating melanoma cells; second, it prevented IL-18-dependent VCAM-1 expression on the hepatic microvasculature which decreased by 50% the microvascular retention of melanoma cells in the liver; and third, it prevented melanoma cell responses to hepatic IL-18, further affecting VLA-4-dependent melanoma cell adhesion and proliferation. Therefore, by acting on host and tumor-dependent responses to IL-18, RVL very efficiently inhibited cancer cell arrest and growth initiation in the tumor microenvironment. Thus, this study uncovers a pathophysiological mechanism accounting for the metastasis-chemoprevention effect of a natural product in the liver.

## Competing interests

The authors declare that they have no competing interests.

## Authors' contributions

CS performed most of *in vitro *and *in vivo *studies and contributed to manuscript preparation; EO contributed to *in vivo *studies and to manuscript preparation; EE, NG, MV and LM contributed to *in vitro *studies; FVV conceived of the study, participated in its design and coordination, and wrote this manuscript. All authors have read and approved the final manuscript.

## Abbreviations

B16M: B16 melanoma; CM: conditioned media; ELISA: enzyme-linked immunosorbent assay; HSE: hepatic sinusoidal endothelium; VEGF: vascular endothelial growth factor; IL-18: interleukin-18; RVL: resveratrol; VCAM-1:vascular cell adhesion molecule-1
